# Characteristic of *Enterococcus faecium* clinical isolates with quinupristin/dalfopristin resistance in China

**DOI:** 10.1186/s12866-016-0863-8

**Published:** 2016-10-21

**Authors:** Shanshan Wang, Yinjuan Guo, Jingnan Lv, Xiuqin Qi, Dan Li, Zengqiang Chen, Xueqing Zhang, Liangxing Wang, Fangyou Yu

**Affiliations:** 1 Department of Laboratory Medicine, the First Affiliated Hospital of Wenzhou Medical University, Wenzhou, 325000 China; 2 Department of Respiratory Medicine, the First Affiliated Hospital of Wenzhou Medical University, Wenzhou, 325000 China

**Keywords:** *Enterococcus faecium*, Quinupristin/dalfopristin, Resistance

## Abstract

**Background:**

Quinupristin/dalfopristin (Q/D) is a valuable alternative antibiotic to vancomycin for the treatment of multi-drug resistant *Enterococcus faecium* infections. However, resistance to Q/D in *E. faecium* clinical isolates and nosocomial dissemination of Q/D-resistant *E. faecium* have been reported in several countries and should be of concern.

**Results:**

From January 2012 to December 2015, 911 *E. faecium* clinical isolates were isolated from various specimens of inpatients at the first Affiliated Hospital of Wenzhou Medical University located in Wenzhou, east China. Of 911 *E. faecium* clinical isolates, 9 (1.0 %, 9/911) were resistant to Q/D, with the Q/D MIC values of 64 mg/L(1), 32 mg/L(1), 16 mg/L(3), 8 mg/L(1) and 4 mg/L(3) determined by broth microdilution. All Q/D-resistant isolates were susceptible to vancomycin, tigecycline and teicoplanin but resistant to penicillin, ampicillin and erythromycin. *vatE* was only found in one Q/D-resistant *E. faecium* isolate while *vatD* was not detected in any of the isolates tested. 8 of 9 Q/D-resistant *E. faecium* isolates were found be positive for both *ermB* and *msrC*. The combinations of Q/D resistance determinants were *ermB*-*msrC* (7 isolates) and *ermB*-*msrC*-*vatE* (one isolate). ST78, ST761, ST94, ST21 and ST323 accounted for 4, 2, 1, 1 and 1 isolate, respectively, among which ST78 was the prevalent ST.

**Conclusion:**

Q/D-resistant *E. faecium* clinical isolates were first described in China. Carriage of *vatE*, *ermB* and *msrC* was responsible for Q/D resistance.

## Background

*Enterococci*, which are the normal commensals in the intestinal tract of humans and animals, are the common cause of nosocomial infections*. Enterococcus faecalis* and *Enterococcus faecium* are responsible for most of enterococcal infections, such as urinary-tract infections, intra-abdominal infections, bacteremia, and endocarditis [1]. With an intrinsic and acquired resistance to some antimicrobial agents, *Enterococci* have become important nosocomial pathogens [2]. Infections caused by multidrug -resistant *Enterococci*, especially multiple resistances to vancomycin, penicillin, and aminoglycoside (high-level resistance), are of a major concern, making enterococcal infections a serious and life-threatening disease [1]. Increase of vancomycin-resistant *Enterococci* (VRE) limits the selection of vancomycin for treatment of enterococcal infections. Therefore, attention has been directed toward the alternatives for the treatment of enterococcal infections, especially VRE infections.

Quinupristin/dalfopristin(Q/D) is a combination of streptogramins B (quinupristin) and streptogramins A (dalfopristin) at 30:70 ratio. Streptogramins A and B are bacteriostatic when used separately but act synergistically when combined. The synergic behavior of the combination results in Q/D rapidly bactericidal against the majority of Gram-positive organisms [3]. Clinically, Q/D is mainly used for the treatment of infections caused by multidrug-resistant Gram-positive *cocci* [4, 5]*.* Q/D is effective against *E. faecium*, but not against *E. faecalis* [6]. As *E. faecalis* isolates possess a chromosomal gene named *lsa* responsible for lincosamide and streptogramin A resistance, which results in all *E. faecalis* with intrinsic resistance to Q/D [2]. Q/D has been successfully used for the treatment of vancomycin-resistant *E. faecium* (VREf) infections [7, 8]. The combination of Q/D and high-dose ampicillin was used successfully for treating persistent bacteremia and endocarditis caused by VREf [5, 9]. Q/D is a valuable alternative to vancomycin for the treatment of multi-drug resistant *E. faecium* infections. However, resistance to Q/D in *E. faecium* clinical isolates and nosocomial dissemination of Q/D-resistant *E. faecium* have been found in several countries [10–13]. Although the prevalence of Q/D resistance among these isolates from humans is still low, emergence and dissemination of Q/D-resistant *E. faecium* limit the therapeutic option of successful treatment of VREf infections. However, Q/D resistance among *E. faecium* isolates from inpatients has not been reported in China. The aim of the present study was to elucidate the prevalence of Q/D resistance among *E. faecium* isolates from the various specimens of inpatients at a tertiary teaching hospital between January 2012 and December 2015.

## Methods

### Collection of clinical isolates and *E. faecium* identification

From January 2012 to December 2015, a total of 911 non-duplicate *E. faecium* isolates (single isolate per patient) from various specimens of inpatients in the first Affiliated Hospital of Wenzhou Medical University located in Wenzhou, east China, were collected consecutively for investigating the prevalence of Q/D resistance. When multiple *E. faecium* isolates were isolated from the same patient, the first isolated strain was included. The included isolates were identified as *E. faecium* using Gram’s stain, catalase test and Vitek-2 microbiology analyzer (bioMe’rieu, Marcy l’Etoile, France). Q/D-resistant isolates were re-identified as *E. faecium* using Matrix-Assisted Laser Desorption/ Ionization Time of Flight Mass Spectrometry (MALDI-TOF-MS) (bioMe’rieux, Marcy l’Etoile, France) and PCR amplifying 16S rRNA gene. *S. aureus* ATCC25923, *Escherichia coli* ATCC25922 and *Pseudomonas aeruginosa* ATCC27853 were used as control strains for identification of bacteria. The Ethics Committee of the first Affiliated Hospital of Wenzhou Medical University exempted this study from review because the present study focused on bacteria.

### Antimicrobial susceptibility testing

The antimicrobial susceptibility testing for *E. faecium* clinical isolates was determined by Vitek-2 microbiology analyzer (bioMe’rieu, Marcy l’Etoile, France) in accordance with the manufactory’s instructions. Q/D resistance initially determined by Vitek-2 microbiology analyzer was reconfirmed using disk diffusion method were according to the guidelines provided by the Clinical and Laboratory Standards Institute (CLSI) [14]. Q/D minimum inhibitory concentration (MIC) values for Q/D-resistant *E. faecium* clinical isolates were determined twice using broth microdilution method operated by two separate operators in accordance with the guidelines recommended by CLSI [14]. Q/D (Synercid, Astellas Pharma, Inc.) was kindly provided by Pro. Nobumichi Kobayashi from Sapporo Medical University (Japan). *S. aureus* ATCC25923, *E. coli* ATCC25922 and *P. aeruginosa* ATCC27853 were used as control strains for antimicrobial susceptibility testing.

### Detection of determinants responsible for Q/D resistance

The genes responsible for resistance to streptogramin B antibiotics including *erm* (*ermA*, *ermB* and *ermC*) and *msrC,* and streptogramin A antibiotics including vat (*vatD* and *vatE*) were detected by PCR assays with specific primers and reaction conditions described previously [15–17]. DNA sequencing was used for the identification of genotype of the genes tested.

### Multi-locus sequence typing (MLST)

MLST of *E. faecium* isolates was performed using amplification of internal fragments of the seven housekeeping genes including *adk* , *atpA* , *ddl* , *gyd*, *gdh*, *purK* and *pstS* of this organism as described previously [18]. Following purification and sequencing of these genes, the sequences were compared with the existing sequences available on the MLST website for *E. faecium* (http://efaecium.mlst.net), and STs were determined according to the allelic profiles.

## Results and discussion

### Prevalence of Q/D resistance among *E. faecium* clinical isolates

Among 911 *E. faecium* clinical isolates over the study period, 9 (1.0 %, 9/911) were resistant to Q/D determined by the Vitek-2 Automated Microbiology Analyzer with GPS card and disk diffusion method. The Q/D MIC values for 9 Q/D-resistant *E. faecium* clinical isolates determined by broth microdilution method were as follows: 64 mg/L, 1; 32 mg/L, 1; 16 mg/L, 3; 8 mg/L, 1 and 4 mg/L, 3 (Table 1). All Q/D-resistant isolates were susceptible to vancomycin, tigecycline and teicoplanin but resistant to penicillin, ampicillin and erythromycin. Eight (88.9 %) of 9 isolates with Q/D resistance were resistant to moxiofloxacin, levofloxacin and ciprofloxacin. Five, 1 and 5 isolates were resistant to gentamicin (high-level resistance), linezolid and nitrofurantoin, respectively (Table 1). As Q/D is not available in China, the patients infected by these Q/D-resistant *E. faecium* isolates were not subject to be treated by Q/D. Therefore, we speculate that the acquisition of Q/D resistance among *E. faecium* clinical isolates in the present study was not associated with the consumption of Q/D. The previous study reported that the extensive in-feed use of virginiamycin showed full cross-resistance with Q/D selected for streptogramin-resistant enterococci and resulted in a reservoir of resistance genes in production animals [19]. Q/D resistance determinants from animal-associated enterococcal isolates can spread to *E. faecium* human isolates [20]. However, in the present study, it remains unknown whether these Q/D-resistant *E. faecium* clinical isolates was indeed associated with the isolates from animals. To our knowledge, the present study is the first report of Q/D resistance among *E. faecium* clinical isolates in China. Up to now, although the prevalence of Q/D resistance among *E. faecium* clinical isolates was very low, the intermediate resistance to Q/D was relatively high. A investigation from Japan reported that none was resistant to Q/D while 28 (17.6 %, 28/159) were intermediate resistant to this antimicrobial agent (MIC = 2 mg/L) among 159 *E. faecium* isolates from clinical specimens in a Japanese hospital from 1997 to 2006 [21]. A study from Greek reported that 250 of 865 (28.9 %) *E. faecium* isolates from patients of eight Greek hospitals between 2005 and2006 were intermediate-resistant to Q/D (MICs = 1.5–4 mg/L) [22]. In another report, all 60 primary clinical isolates of *E. faecium* with resistance to glycopeptides were fully susceptible to Q/D, with MIC50 and MIC90 values of 1.0 mg/L and 1.5 mg/L [23]. These previous studies and our study support the evidence that Q/D is still an effective and valuable antimicrobial agent for treating infections caused by multi-resistant *E. faecium*, even VREf. Nevertheless, the emergence of Q/D resistance, especially increased intermediate resistance to this antimicrobial agent, has become a concern. Moreover, a high prevalence (10.0 %) of Q/D resistance among *E. faecium* clinical isolates was found in Korea, which was associated with both clonal spread and the sporadic emergence of Q/D-resistant isolates [12]. Hsueh et al. also reported a similar high prevalence (9 %) of Q/D resistance among vancomycin-resistan*t E. faefium* clinical isolates in Taiwan [24]. As our study did not investigate the prevalence of intermediate resistance to Q/D among *E. faecium* clinical isolates, it remains unclear whether there was a trend of increase in intermediate resistance to Q/D, which should be further investigated.

**Table 1:** The characteristics of Q/D-resistant *E. faecium* isolates

No.	Specimen	ST	Q/D MIC value (mg/l)	Antimicrobial resistance profile^a^	Antimicrobial susceptibility profile^a^	Q/D resistance determinants
1	pus	78	16	MOX, GEN(H), P, E, LZD, F, LEV, AMP, CIP	TEC, TGC, TET, VAN	*ermB, msrC*
2	urine	78	32	MOX, GEN(H), P, E, LEV, AMP, CIP	LZD, TEC, F, TGC, TET, VAN	*ermB, msrC*
3	catheter	94	16	GEN(H), P, E, LEV, AMP, CIP,	MOX, LZD, TEC, F, TGC, TET, VAN	
4	exudate	761	8	P, E, AMP	LEV, CIP, MOX, GEN(H), TEC, F, TGC, TET, VAN, LZD	*ermB, msrC*
5	wound	21	64	MOX, GEN(H), P, E, F, LEV, AMP, CIP	LZD, TEC, TGC, TET, VAN	*ermB, msrC, vatE*
6	exudate	323	4	MOX, P, E, F, LEV, AMP, CIP	GEN(H), LZD, TEC, TGC, TET, VAN	*ermB, msrC*
7	urine	78	4	MOX, P, E, LEV, AMP, CIP	GEN(H), LZD, F, TEC, TGC, TET, VAN	*ermB, msrC*
8	blood	761	16	MOX, P, E, LEV, AMP, CIP, TET, F	GEN(H), LZD, TEC, TGC, VAN	*ermB, msrC*
9	tissue	78	4	MOX, GEN(H), P, E, LEV, AMP, CIP, F	LZD, TEC, TGC, TET, VAN	*ermB, msrC*

^a^ *P* penicillin, *AMP* ampicillin, *E* erythromycin, *TET* tetracycline, *TGC* tigecycline. *GEN(H)* high-level gentamicin, *CIP* ciprofloxacin, *MOX* moxifloxacin, *LEV* levofloxacin, *F* nitrofurantoin, *LZD* linezolid, *VAN* vancomycin, *TEC* teicoplanin

### Determinants of Q/D resistance among *E. faecium* clinical isolates

The resistance to Q/D is associated with enzymatic modification of the antibiotic, active transport or efflux mediated by an ATP-binding protein, and alteration of the target site [6]. Both resistance to streptogramin A and streptogramin B is necessary for occurrence of Q/D resistance [25]. Carriage of more than one streptogramin A resistance gene *(vat* or *vga)* is necessary for the occurrence of Q/D resistance in an organism [6]. The streptogramin A resistance genes found in *E. faecium*are were *vatD* and *vatE* encoding acetyltransferases [6]. Modification of dalfopristin by the acetyltransferases VatD and VatE renders it ineffective, abolishing the synergy with quinupristin. *vatD* was initially found in an *E. faecium* isolate from a hospitalized patient in Europe [26].*vatD* and *vatE* have been found in *E. faecium* animal and human isolates in Europe, USA and Korea [6, 27, 28]. In contrast, some studies reported that none of the genes involved in the expression of dalfopristin resistance (*vatD*, *vatE*, *vgaA* and *vgaB*) were found in any Q/D-intermediate-resistant or -resistant *E. faecium* isolates [21, 22, 27]. Similarly, in the present study, *vatE* was only found in one Q/D-resistant *E. faecium* isolate while *vatD* was not detected in any of the isolates tested (Table 1). The most commonly known resistance to streptogramin B in *enterococci* is the macrolides, lincosamides, and streptogramins B ( MLSB) resistance conferred by the *erm* genes (*ermA*, *ermB* and *ermC*) encoding an enzyme that dimethylates an adenine residue in the 23S rRNA, which results in decreased binding of these antimicrobial agents [6]. In the present study, *ermB* was found among 8 of 9 Q/D-resistant *E. faecium* isolates. *vgbA* encoding a streptogramin-inactivating enzyme (lyase) has been found rarely in the isolates of *E. faecium,* but *msrC* conferring resistance to streptogramin B antibiotics by active transport is commonly found in *E. faecium* isolates [29]. In the present study, 8 of 9 Q/D-resistant *E. faecium* isolates were found be positive for *msrC*. The remaining one isolate was negative for all resistance genes tested. Currently, there has not been reported the combination of multiple streptogramin A resistance genes in *E. faecium*. However, combinations of the *vatD-vgbA* and *ermB-vatD* or *vatE* genes were found in Q/D-resistant isolates [10, 30, 31]. The *vatE* was more common than *vatD* in animal sources [11, 28, 32]. In the present study, the combinations of Q/D resistance determinants were *ermB*-*msrC* (7 isolates) and *ermB*-*msrC*-*vatE* (one isolate).

### Molecular characteristics of Q/D-resistant*E. Faecium* clinical isolates

The lineage clonal complex 17 (CC17) (ST17, ST18, and ST78) significantly associated with hospital infections emerges as the high-risk clone responsible for the worldwide spread of VERf [33]. A report from Poland showed the domination of representatives of lineages ST78 and ST17/18 (52.7 and 46.4 %, respectively) among consecutive *E. faecium* clinical isolates collected in 30 hospitals between May 2010 and June 2011 through prospective surveillance in Poland [34]. VERf isolates from 10 infected and 40 colonized inpatients from a single hospital in the north of Spain were assigned to ST17 by MLST [35]. In China, ST78 was the predominant MLST type among VERf clinical isolates [36]. However, the molecular characteristic of Q/D-resistant *E. faecium* clinical isolates is limited. Among 25 Q/D-resistant *E. faecium* isolates from Korea, 10, 9 and 4 belonged to ST78, ST192 and ST203, respectively, with ST78 being the prevalent ST [12]. In the present study, among 9 Q/D-resistant isolates, ST78, 761, 94, 21 and 323 accounted for 4, 2, 1, 1 and 1 isolate, respectively, with ST78 being the prevalent ST (Table 1). In another report, 5 STs including four STs of CC17 were identified in Q/D-intermediate resistant *E. faecium* clinical isolates [21]. In addition, only a VERf isolate from China was found to belong to ST323 [37]. The present study is the second report of an *E. faecium* ST323 isolate with Q/D resistance associated with bloodstream infection.

## Conclusion

In the present study, Q/D-resistant *E. faecium* clinical isolates were first described in China. We also identified that carriage of *vatE*, *ermB* and *msrC* was responsible for Q/D resistance in *E. faecium* clinical isolates.

## Abbreviations

CLSI: Clinical and Laboratory Standards Institute
MALDI-TOF-MS: Matrix-Assisted Laser Desorption/ Ionization Time of Flight Mass Spectrometry
MIC: Minimum inhibitory concentration
MLST: Multi-locus sequence typing
Q/D: Quinupristin/dalfopristin
VREf: Vancomycin-resistant *Enterococcus faecium*
